# A Novel DNA‐Based Dual‐Mode Data Storage System with Interrelated Concise and Detailed Data

**DOI:** 10.1002/smsc.202400094

**Published:** 2024-08-19

**Authors:** Ben Pei, Yongsen Zhou, Yu Yang, Jiaxiang Ma, Rangli Cao, Wen Huang, Liliang Ouyang, Shengli Mi, Zhuo Xiong

**Affiliations:** ^1^ Biomanufacturing Center, Department of Mechanical Engineering Tsinghua University Beijing 100084 China; ^2^ Biomanufacturing and Rapid Forming Technology Key Laboratory of Beijing Beijing 100084 China; ^3^ Biomanufacturing and Engineering Living Systems Innovation International Talents Base (111 Base) Beijing 100084 China; ^4^ Bio‐manufacturing Engineering Laboratory, Tsinghua Shenzhen International Graduate School Tsinghua University Shenzhen Guangdong 518055 China

**Keywords:** DNA‐based data storage, dual‐mode storage system, nanodot array, scanning probe lithography

## Abstract

DNA has emerged as a promising storage medium to meet the soaring need for archival data storage because of its exceptional storage density and stability. However, current DNA‐based data storage systems are incompetent of achieving high‐quality random multiplexed access and frequently accessed data storage, which impedes its practical applications. Here, a dual‐mode storage system is proposed that combines DNA‐based archival data and nanodot‐based active data. This novel data‐storage system is constructed by writing the active and archival data on the same substrate through a facile two‐step process involving scanning probe lithography (SPL), DNA synthesis, and chemical immobilization. The data files are categorized and stored orderly in different microregions of the substrate to achieve efficient random access. On each microregion, the nanodot array stores not only the concise information for the archival DNA data but also contains the corresponding primer sequence. Such interrelation between active and archival data allows for facilely data reading by efficient microscopic modalities and in situ polymerase chain reaction (PCR). Facilitated by the integration of nanodot and DNA, this novel dual‐mode storage system demonstrates efficient data access and the potential of excellent storing capacity, paving the way for the advancement of DNA‐based data storage.

## Graphical abstract

This study proposes a dual‐mode data storage system, which combines nanodot‐based frequently accessed data (concise data) and DNA‐based archival data (detailed data). Concise data writing/reading is achieved through scanning probe lithography (SPL)/microscopy, and detailed data writing/reading is achieved through DNA immobilization and in situ polymerase chain reaction (PCR). This system accomplishes efficient data access, and provides a new management idea.

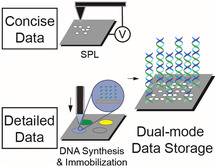

## 1 Introduction

The advent of digitalization has resulted in the exponential growth of information, and conventional storage media is no longer capable of achieving effective and reliable data storage, thus the development of new storage media is in desperate need.^[^ ^1^ ^]^ DNA has emerged as a promising storage medium owing to its remarkable attributes such as high information storage density, extended storage lifespan, and minimal energy consumption.^[^ ^2^ ^]^ However, the development of DNA‐based data storage has long been plagued by the expensive and time‐consuming synthesis and sequencing processes. These issues have been gradually addressed by the development of high‐throughput DNA synthesis,^[^ ^3^ ^]^ enzymatic DNA synthesis,^[^ ^4^ ^]^ next‐generation sequencing, and nanopore sequencing technologies,^[^ ^5^ ^]^ which greatly advanced the DNA‐based data storage from concept to feasibility.^[^ ^6^ ^]^ To further propel the DNA‐based data storage from feasibility to application, the performance of random multiplexed access as well as facile and efficient access of information needs to be greatly improved.^[^ ^7^ ^]^

In practical applications, the target information needs to be selectively retrieved from the complex pool of DNA files. To this end, current DNA‐based storage systems mainly rely on complicated and orthogonal primer pairs for the polymerase chain reaction (PCR)‐based random access of data.^[^ ^8^ ^]^ However, it is practically challenging to design orthogonal primer pairs to eliminate molecular crosstalk, particularly for large‐scale data storage.^[^ ^9^ ^]^ Moreover, the inherent trade‐off between primer length and data payload makes the data storage density and efficiency of random access incompatible with each other.^[^ ^10^ ^]^ Additionally, a small fraction of DNA molecules is irreversibly consumed during the PCR‐based random access, and the PCR‐based amplification of DNA molecules is required to recover the DNA pool.^[^ ^9^, ^11^ ^]^ However, the differences in DNA molecule length, sequence, guanine‐cytosine content make the PCR process uncontrollable, resulting in uneven amplification of different DNA molecules.^[^ ^12^ ^]^ Such PCR bias would gradually accumulate after multiple repetitions, which leads to difficulty in sequencing and even data loss.^[^ ^13^ ^]^ To address these issues, an effective strategy is to construct physically isolated data management systems where different DNA files are separated from each other to facilitate individual access and amplification.^[^ ^14^ ^]^ Various methods have been developed to circumvent the problems associated with PCR‐based random access including emulsion PCR,^[^ ^15^ ^]^ microcapsules,^[^ ^9^ ^]^ and silica microspheres.^[^ ^16^ ^]^

Prior to the processes of PCR and sequencing of DNA, the information of primer sequence needs to be provided. In the process of accessing any data in the DNA pool, the indexing data containing primer sequences is accessed first. As a result, this indexing data is much more frequently accessed than the main data and the process is time‐consuming. To ensure efficient and accurate accessing of the indexing data, a proper medium that allows frequent access should be developed. To this end, Choi et al. have proposed a method of storing primer sequences in a QR‐coded micro‐sized disk embedded with DNA molecules.^[^ ^13^ ^]^ This pioneering work has also achieved, for the first time, the physical connection of indexing data and main data. In practical applications, some specific data is also frequently accessed in addition to the indexing data. These frequently accessed active data could be referred as concise data, while the less‐frequently accessed archival data could be referred as detailed data. However, DNA‐based storage systems with interrelated concise and detailed data have rarely been developed before.

Here, a dual‐mode storage system that combines DNA storage technology and nanodot array storage technology is proposed. This novel system stores detailed data by covalently immobilizing DNA molecules on a solid substrate and concise data in nanodot arrays atop. Moreover, both data files are orderly stored in the different microregions of the substrate. Each microregion contains a detailed file and its corresponding concise file, and a direct bridge between them is constructed by encoding the index of detailed data in the nanodot array. The nanodot array can be readily and efficiently constructed on a solid substrate and imaged through scanning probe lithography (SPL) and atomic force microscopy (AFM), meeting the demands for highly frequent write and read of concise data. Since the DNA molecules are immobilized on the numbered microregion, random multiplexed access to the detailed data can be efficiently and facilely achieved through in situ PCR, facilitating its large‐scale applications. This dual‐mode storage system reconciles efficient access to concise data and the high‐capacity storage of detailed data.

## 2 Results and Discussion

### 2.1 Design and Mechanism of the Dual‐Mode Data Storage System

**Figure 1:**
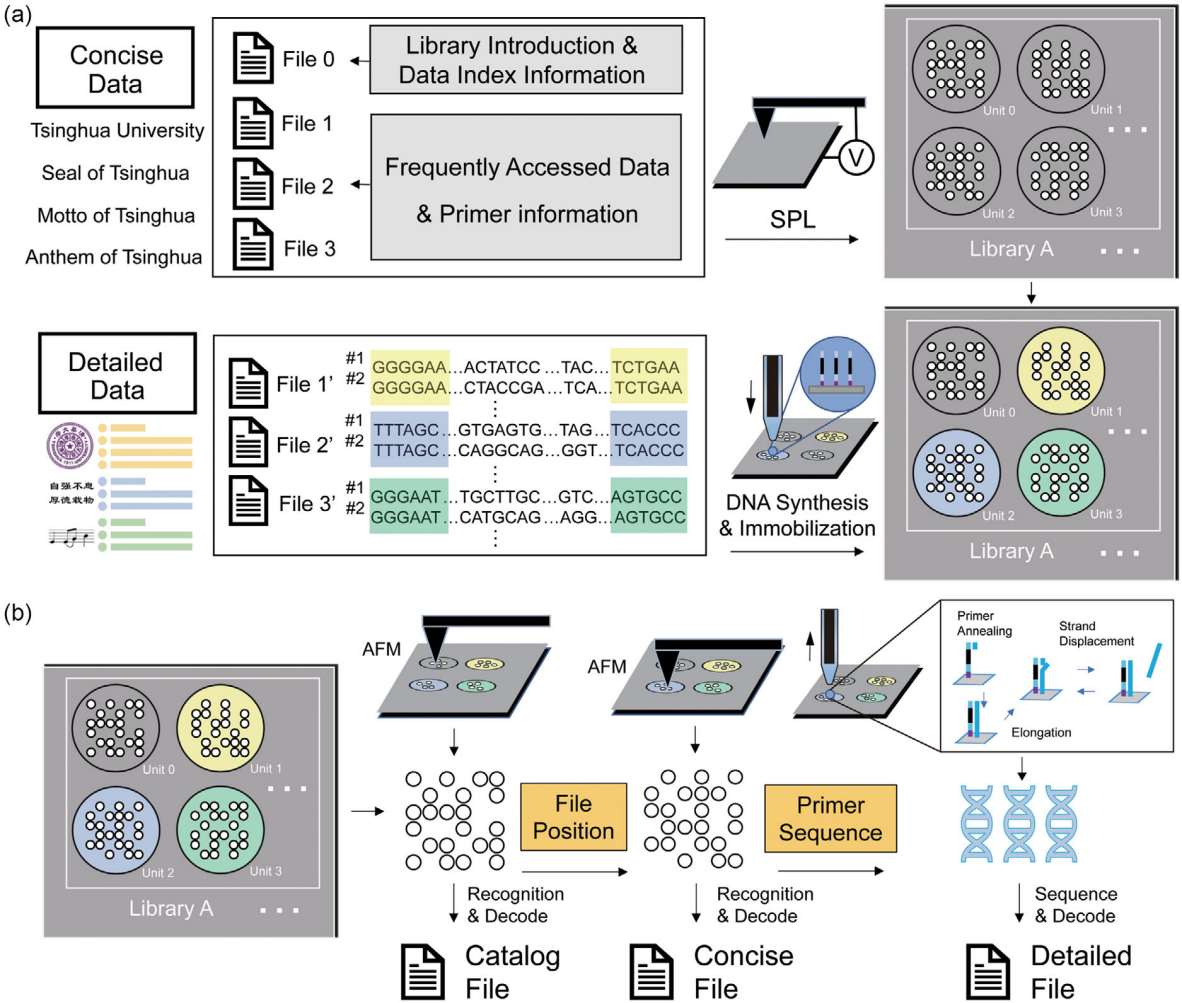
Illustration of the workflow for the dual‐mode data storage system. a) The fabrication of the dual‐mode data storage system starts with the categorization and encoding of library, followed by data writing involving SPL, DNA synthesis, and immobilization. b) The reading process of the dual‐mode data storage system starts with the access of catalog file by AFM, followed by accessing the concise files with the positional information obtained from the catalog file. The detailed files of interest are accessed by in situ PCR and sequencing whose primer sequences are provided by the corresponding concise files.

To achieve efficient management of concise and detailed data, the original library is first categorized and encoded according to the access frequency (**Figure** **1**a). Specifically, the concise data containing two different types of files (i.e., catalog file and concise files) are converted to binary files, which are written on a solid substrate in the form of physically separated microregions by the well‐established SPL. The catalog file briefly introduces the whole library and includes the location of concise files. The concise files consist of the frequently accessed data and primer sequences information of detailed data. To write the detailed data, relevant detailed files are converted to binary files and then DNA sequences, followed by covalent immobilization on the substrate. In a typical library, the concise data refers to one catalog file located in unit 0 and three concise files (i.e., the introduction of the seal, motto, and anthem of Tsinghua University) located in other units, while the detailed data refers to detailed files (i.e., the pattern or detail content of the seal, motto, and anthem of Tsinghua University). Meanwhile, a concise file and its paired detailed file are stored in a same unit. In this work, concise data refers to files with high frequency of access, which are stored on a substrate in the form of nanodot array. Detailed data refers to files with less frequency of access, which are stored in DNA molecules. Each detailed file is correlated with the corresponding concise file, and the DNA molecules are immobilized at the same location of the corresponding nanodot array.

The data reading process of this dual‐mode storage system is shown in Figure 1b, which starts with the acquisition of catalog file by scanning unit 0. With the obtained positional information, the concise file of interest can be easily located and accessed by morphological scanning techniques (e.g., AFM). In scenarios where more detailed information is required, the primer sequence embedded in the concise file can be read, allowing the corresponding detailed file to be obtained through in situ PCR and sequencing. Since the concise data is encoded in the nanodot array, high data density and straightforward access are warranted. The interrelation between concise and detailed data coupled with the covalent immobilization of DNA molecules enables efficient random multiplexed access.

### 2.2 The Data Writing Process through SPL and DNA Immobilization

**Figure 2:**
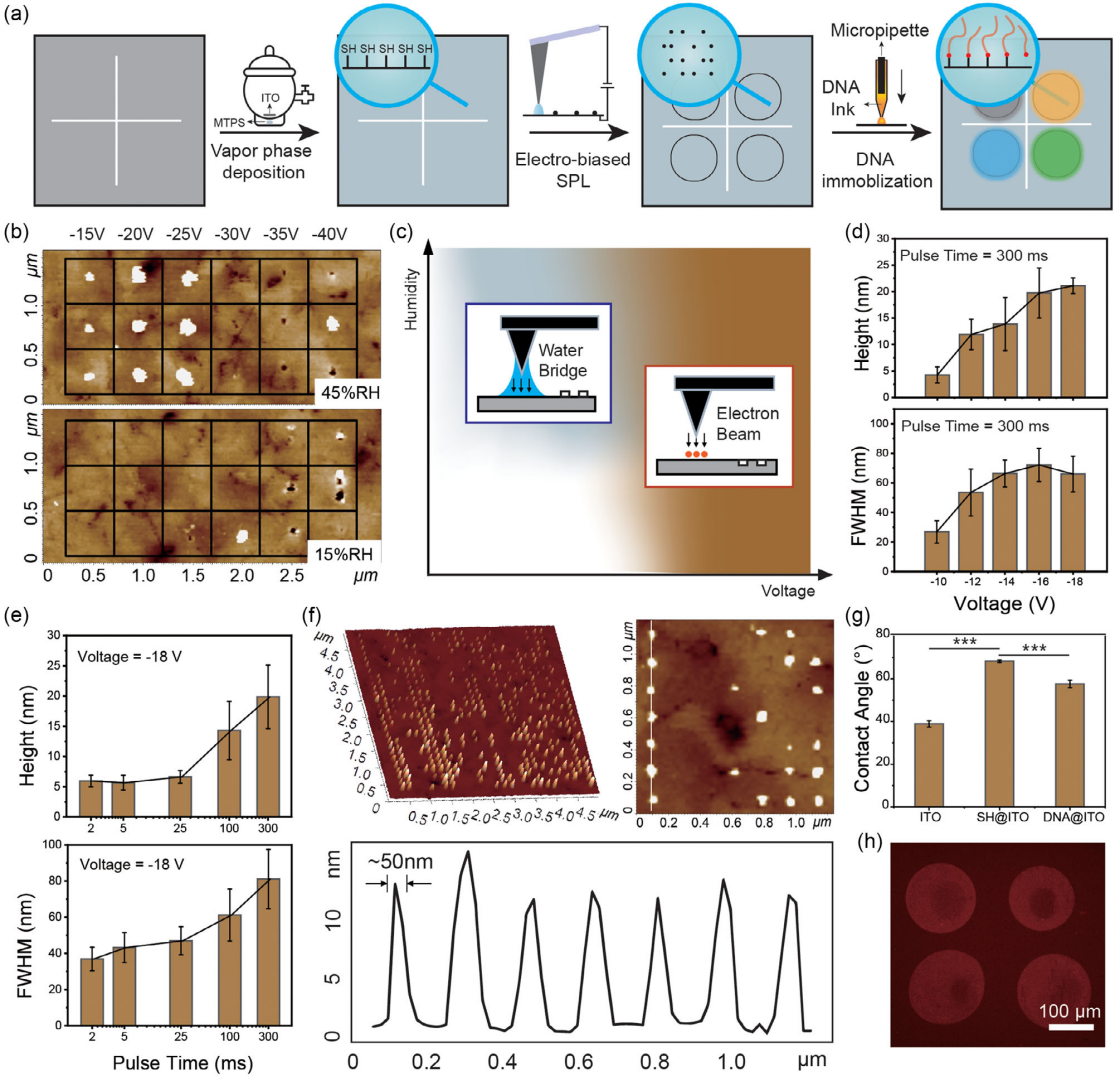
Mechanism and characterization of data writing. a) Illustration of the data writing process, which includes silanization, nanodot array fabrication, and DNA immobilization. b) The lithography result at different voltage and humidity. c) Illustration of different dominant working mechanism under the process of fabricating nanostructures at different voltage and humidity. d,e) The influence of voltage magnitude, pulse time on nanodot array fabrication (Data presented as mean ± SD, *n* = 3). f) Morphological characterization of the nanodot array under optimized condition. g) Wetting characterization of the substrate at different state (Data presented as mean ± SD, *n* = 5, *P*‐values are calculated using *t*‐test, ***P < 0.001, *p* value between ITO and SH@ITO is 2.9 × 10^−10^, *p* value between SH@ITO and DNA@ITO is 2.3 × 10^−6^). h) Fluorescence characterization of the immobilized DNA.

Constructing dual‐mode data storage system mainly involves the concise and detailed data writing through the nanodot array fabrication by SPL and the following DNA immobilization by a micropipette (**Figure** **2**a). To ensure the random multiplexed access of detailed data by in situ PCR, covalent immobilization of DNA molecules is desired. Among various DNA immobilization chemistries, the thiol‐disulfide exchange reaction is an efficient and reliable option to achieve robust covalent anchoring of DNA on solid substrates owing to its specificity and minimized side reactions.^[^ ^17^ ^]^ In a typical process of DNA immobilization, a pristine substrate is first cleaned and modified with thiol groups by vapor phase deposition of 3‐mercaptopropyl silane (MTPS) at room temperature. Meanwhile, the single stranded DNA (ssDNA) containing detailed data is modified with disulfide groups at the 5′ end. To confine the DNA immobilization in a microregion, tens of nanoliters of DNA solution is applied to the target location by micropipette with the aid of microscope. The DNA molecules not immobilized on the substrate are washed off by a specific buffer solution. As to the writing of concise data, the SPL technique is employed owing to its advantages of high resolution and ambient working condition.^[^ ^18^ ^]^ Here, the robust and well‐established electro‐biased SPL is used to fabricate the nanodot array by altering the physiochemical properties of conductive substrates in the presence of high electric field.^[^ ^19^ ^]^ Meanwhile, for better observation, a transparent substrate is preferred. Therefore, the indium tin oxide (ITO) glass is selected as the substrate for the construction of dual‐mode data storage system. While there has been extensive research on electro‐biased SPL, there is still a lack of SPL works for the high‐quality construction of nanodots on ITO.^[^ ^20^, ^21^, ^22^ ^]^ Our work achieves high‐precision, aspect ratio nanodot construction on SPL and deeply analyzes the mechanism.

Previously, it was reported that applying a bias voltage between the tip of SPL and ITO surface would generate protruded lines of about 100 nm through anode oxidation reactions at the water bridged tip–ITO interface.^[^ ^23^ ^]^ To achieve data storage and improve the density, nanodots are preferred over nanolines. Therefore, the electro‐biased SPL was optimized to fabricate nanodots by investigating key influencing factors including humidity, voltage, and pulse time. As shown in Figure 2b, when the humidity was low (15% RH), only uncontrollable nanodots were produced with the increase of voltage. Under high humidity (45% RH), and the generation of nanodots was dependent on the voltage. When the voltage was below −25 V, the nanodots were stable, whereas nanodots became unstable and would occur as pits when the voltage was above −25 V. These findings suggested the importance of water bridge in producing stable protruded structures. At low humidity levels, the formation of water bridge is challenging, impeding the peroxidation process. When humidity is suitable, but voltage is higher than a threshold, the elevated current would disrupt the stability of water bridge, hindering the peroxidation process. It is hypothesized that a field emission effect is dominated over the anode oxidation at higher voltage.^[^ ^24^ ^]^ The high energy electron beam emitted from the tip would decompose the ITO surface, resulting in the formation of nano‐sized pits. The involving working mechanism of fabricating nanostructures at different voltage and humidity is diagramed in Figure 2c. Next, the effects of voltage and pulse time on the morphological features of nanodots were investigated at the humidity of 45% RH. Figure 2d shows the change of height and full width at half maximum (FWHM) of nanodots at different voltages under a pulse time of 300 ms (Figure S1, Table S1). It was observed that the height and FWHM increased with the increasing of voltage. Similarly, the height and FWHM showed a gradual increase with the increasing of pulse time at a fixed voltage of –18 V (Figure 2e, Figure S1, Table S2). This is because higher voltage and longer pulse time correspond to higher energy, leading to an intense anode oxidation and hence higher height and FWHM. It was also observed that, the effect of voltage on the height and FWHM of nanodot was more significant than that of pulse time. After the optimization, a voltage of −15 V, pulse time of 20 ms, and a 45% RH were employed to fabricate the encoded nanodot array. As shown in Figure 2f, a 28 × 28 dot array with 50 nm FWHM and 10 nm height can be clearly observed by AFM, indicating the feasibility and high fidelity of the electro‐biased SPL in encoding concise data. Meanwhile, such dot array was produced within 1 min, highlighting the efficiency of concise data writing by the electro‐biased SPL.

To verify the immobilization of DNA molecules, the surface wettability of ITO glass at different state was first examined. As shown in Figure 2g, the pristine ITO exhibited a contact angle of 39°, which increased to 68° after thiolation, indicating the successful modification of ITO substrate (Table S3). The increased hydrophobicity could greatly facilitate the confinement of DNA droplets to predetermined locations, allowing for the accurate DNA immobilization in microregions. After DNA immobilization, the contact angle dropped, suggesting that the hydrophilic DNA molecules have been successfully bonded to the substrate. To ensure the effectiveness of DNA immobilization, fluorescent‐labeled and disulfide‐modified DNA was reacted with thiol‐modified glass substrate. As shown in Figure 2h, the microregions with immobilized DNA exhibited obvious red fluorescence compared to the background. Noted that the ITO substrate was not used because the conductivity could quench the emission of fluorophores on DNA.^[^ ^25^ ^]^

### 2.3 The Process of Reading Data through AFM and In situ PCR

**Figure 3:**
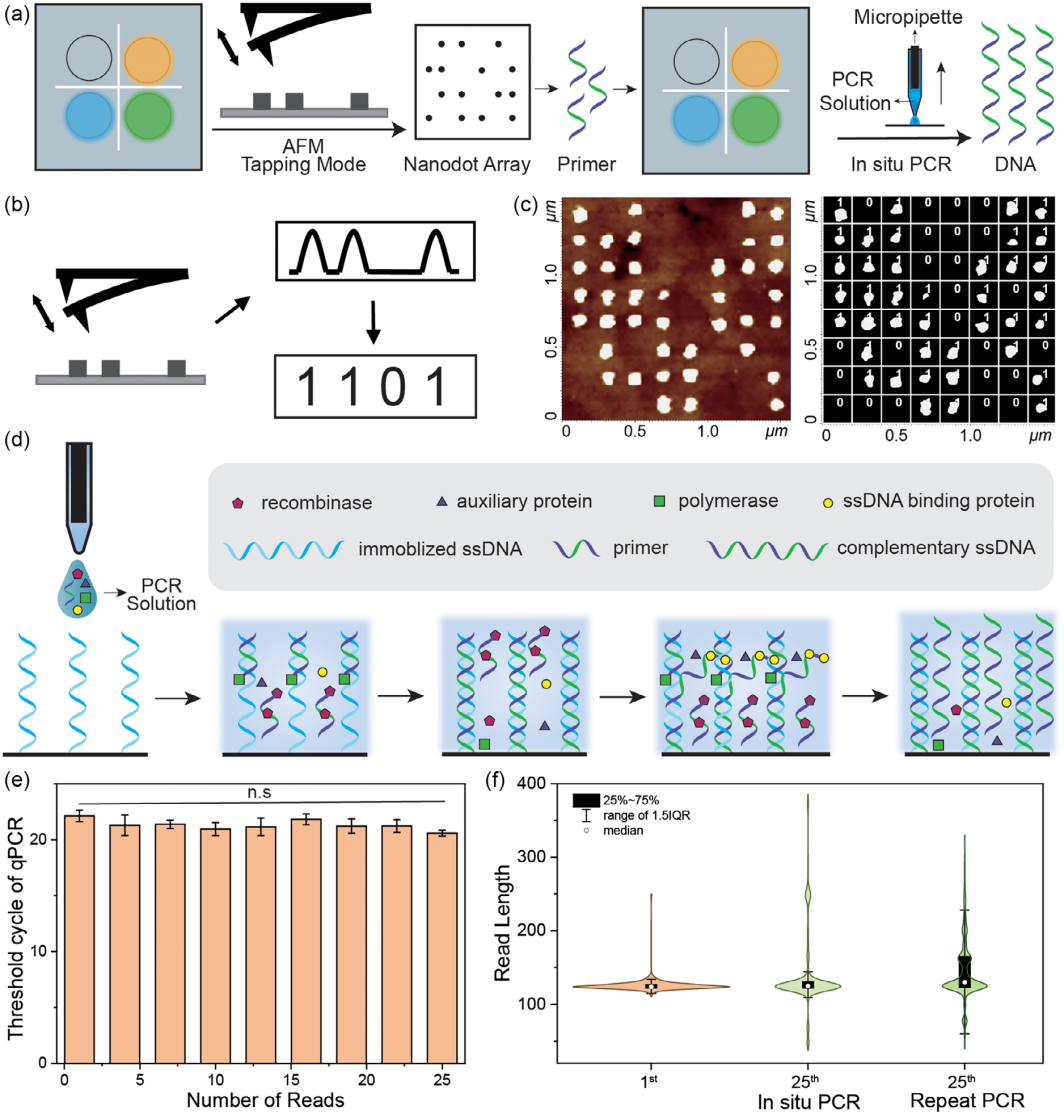
Mechanism and characterization of data reading. a) Illustration of the data writing process which includes AFM scanning and DNA in situ PCR. b) Illustration of the process of nanodot array reading by AFM. c) A typical example of the morphological image and recognition of nanodot array. d) Illustration of the process of in situ isothermal PCR. e) The threshold cycle of qPCR for DNA molecules obtained from 25 repeated extractions (Data presented as mean ± SD, *n* = 5, *P*‐values are calculated using one‐way ANOVA, *p* = 0.1635). f) Comparison of the sequencing read length distribution results of in situ PCR and conventional repeat PCR.

To read the target information in the dual‐mode data storage system, the concise data is first accessed by scanning the nanodot array with AFM. In most cases, target information can be found in the concise data. If not, with the primer information obtained from the concise data, the required detailed data can be easily accessed by in situ PCR (**Figure** **3**a). As illustrated in Figure 3b, under the tapping mode, the morphological features on the ITO surface were recorded as height curves, where the protruded and flatten parts refer to digit “1” and “0”, respectively. Figure 3c shows a typical AFM height image and its recognized result after binary processing. It was observed that all the protruded features were successfully converted to “1”, indicating the excellent reading performance of concise data by AFM. Moreover, the non‐destructive acquisition of nanodot array by AFM tapping mode facilitates the multiple access of the concise data.

When detailed information is required, access to the detailed data can be facilely achieved by in situ PCR (Figure 3d). Since the PCR solution must be confined to microregions for random access, the required volume is small and thus highly affected by temperature. Therefore, an isothermal PCR was employed at room temperature. Specially, the surface immobilized ssDNA is converted to double strands and the as‐generated complementary ssDNA is released by displacement in the presence of recombinase, polymerase, ssDNA binding protein, auxiliary protein, primer, and then subjected to sequencing. This method for DNA access is gentler, and allowed for in situ access compared to the conventional way. To evaluate the repeatable access of detailed data, the in situ PCR was performed on the same microregion for multiple times and the as‐generated complementary ssDNA was analyzed by quantitative real‐time polymerase chain reaction (qPCR). As shown in Figure 3e (Figure S2, Table S4), the threshold cycles of qPCR did not show significant increase within 25 reads, suggesting that the amount of surface‐bonded ssDNA was well maintained during multiple access. The 25th read was further subject to sequencing to assess the preservation of original characteristics. Compared to conventional repeat PCR, the sequencing read length of in situ PCR was much more centralized as indicated by smaller interquartile range (Figure 3f, Figure S3), highlighting the better preservation and improved repeatable data reading performance of in situ PCR.

In conventional DNA data storage, if the index information is stored in a DNA pool, it will take several hours of PCR and sequencing to obtain it. But in the dual‐mode storage system, it only takes a few minutes of AFM scanning to obtain it. On the contrary, the dual‐mode storage system allows for easy access to frequently needed concise data through AFM. In this case, when reading the concise data stored in dot array, the data is available in a few minutes of AFM scanning, instead of hours as in the case of conventional DNA data storage. When reading the detailed data, it is necessary to first obtain the index through AFM scanning, and then retrieve the detailed data via in situ PCR and sequencing. For the overall storage system, the read/write process involves special equipment and laborious techniques such as deposition, sample preparation, SPL, image processing, and finally, PCR. Although these operations may result in increased time and resource consumption when reading DNA data, the system provides well management of DNA data, allowing for multiple random reads. The frequently accessed concise data is well‐integrated with the associated detailed data and can be conveniently read through AFM scanning. Thus, while the dual‐mode storage system does not directly reduce the time or cost of reading, it introduces a new management system in DNA data storage, enabling on‐demand access to indexing data and DNA molecules with minimal damage.

Furthermore, although AFM does not read data as fast as traditional memory devices, it has the significant advantage that it can be easily implemented without the need for complex biochemical manipulation. Recent studies also show that the speed of AFM imaging is increasing,^[^ ^26^ ^]^ with current high‐speed AFM technology already capable of scanning an image with 200 × 200 pixels in 1 s.^[^ ^27^ ^]^ These advances demonstrate the speed and potential for speed‐up of AFM.

### 2.4 Data Density and Stability of the Dual‐Mode Data Storage System

**Figure 4:**
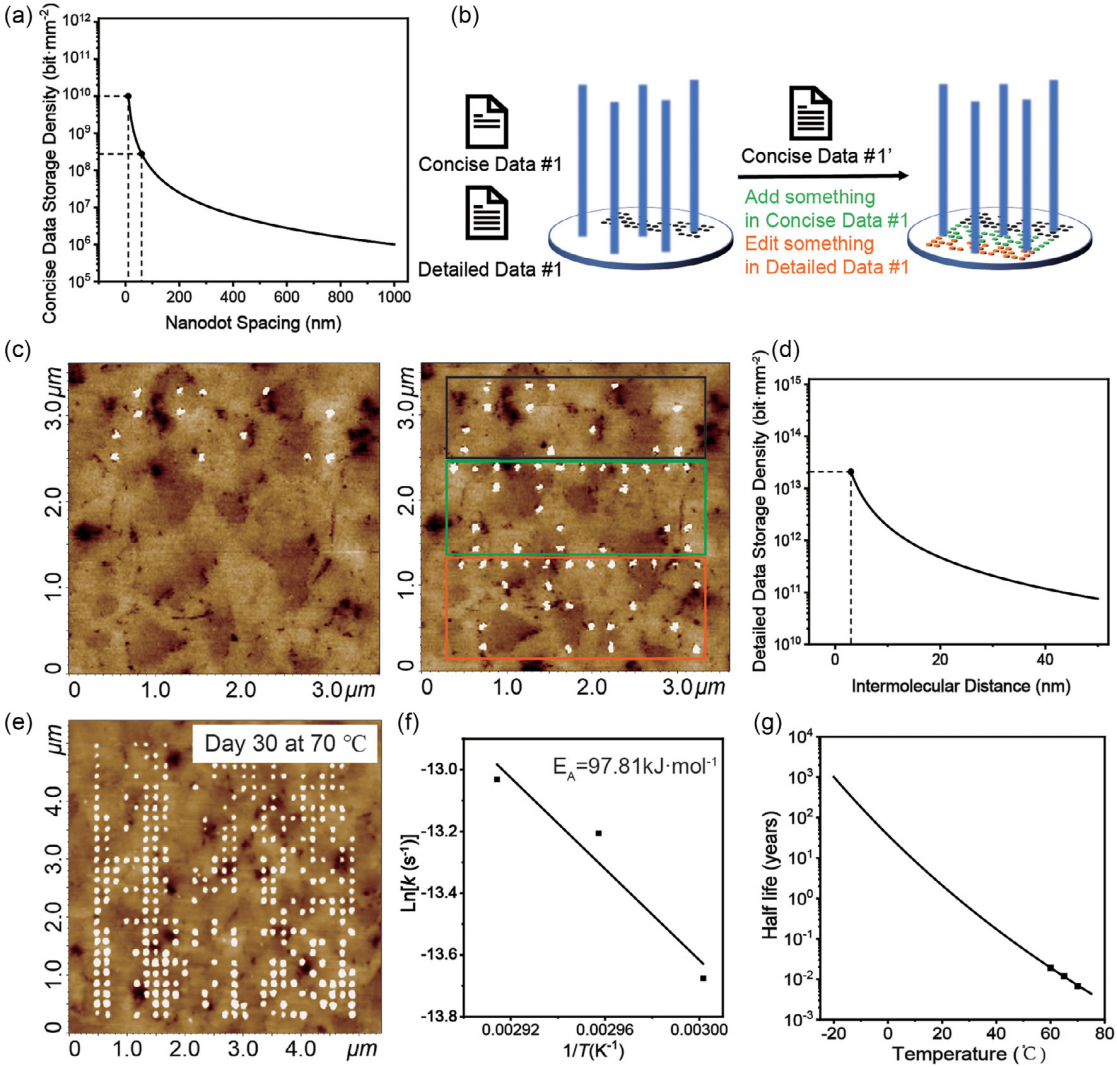
Data density and stability of the dual‐mode data storage system. a) Theoretical calculation of storage density of concise data. b,c) Illustration and demonstration of data editing with supplemented nanodot array. d) Theoretical calculation of storage density of detailed data. e) The morphological stability of nanodot array at a temperature of 70 °C. f,g) Analysis of activation energy and the extrapolated half‐life of detailed data.

Next, the data density and stability of the dual‐mode storage system were evaluated. To calculate the density of concise data, a theoretical density curve was obtained from the equation *ρ* = *d* ^−2^ (**Figure** **4**a), where *d* refers to the spacing between two neighboring nanodots (Note 1). Since the FWHM of nanodot is about 50 nm, the maximum density could reach 3 × 10^8^ bit mm^−2^. In fact, previous studies have demonstrated that the size of nanodots could be further decreased to 10 nm by SPL,^[^ ^28^ ^]^ corresponding to a storage density of about 7 × 10^9^ bit mm^−2^. Such storage density is comparable to hard drives.^[^ ^29^ ^]^ Since the nanodot array takes only a small fraction of the microregion, the remaining space could be used for further data writing (Figure 4b,c). This practical feature allows for the supplement of concise data and revisal of detailed data. Theoretically, the density of detailed data can be calculated by the equation *ρ* = *L* × *d* ^−2^, where *L* is the total amount of data per DNA molecule and *d* is the spacing between two neighboring DNA molecules. Although d is difficult to be accurately measured, an approximate value could be obtained from a previous study.^[^ ^30^ ^]^ Assign *d* = 10 nm, the detailed data density of this dual‐mode storage system could reach 10^12^ bit mm^−2^ (Figure 4d). Studies have shown that very small intermolecular distance can be achieved with framework nucleic acids, and the limit is about 3–5 nm.^[^ ^31^, ^32^ ^]^ Based on this, the achievable data storage density limit is calculated to be about 2 × 10^13^ bit mm^−2^. Although it is lower than conventional DNA storage, it is also significantly higher than traditional storage media.^[^ ^33^ ^]^

In the experiments, the dual‐mode storage system was preserved in a dry nitrogen environment. The stability of concise data was assessed by exposing the storage system to high temperatures and acquiring the AFM images at different time intervals (Figure S4). It was found that the nanodots were still clear and complete after 1‐month storing at 70 °C (Figure 4e). In addition to the high temperature stability, the nanodot array was stable against the fouling of contaminants from the environment, which could be realized by a simple washing process (Figure S5). To assess the stability of detailed data, accelerated aging experiments were conducted at various temperatures. Specifically, according to the Arrhenius equation, the activation energy was first derived to be 97.81 kJ mol^−1^ (Figure 4f, Figure S6, Table S5, Note 2). This value is lower than that of DNA (120–155 kJ mol^−1^) because the surface‐bonded DNA could also break at the bonding interface in addition to the chain itself.^[^ ^34^ ^]^ Next, the half‐life of detailed data at different temperatures was calculated and their relationship was fitted and plotted in Figure 4g. At a temperature of 0 °C, the half‐life of detailed data could reach 40 years. DNA molecules were sequenced after one decay half‐life. The sequencing results indicate that the error rate of the DNA molecules was elevated after accelerated aging, but it remained within acceptable limits (Figure S7 and S8). Although a small number of DNA strands were not correctly recovered, most of the strands were successfully retrieved.

### 2.5 Demonstration of Dual‐Mode Data Storage System

**Figure 5:**
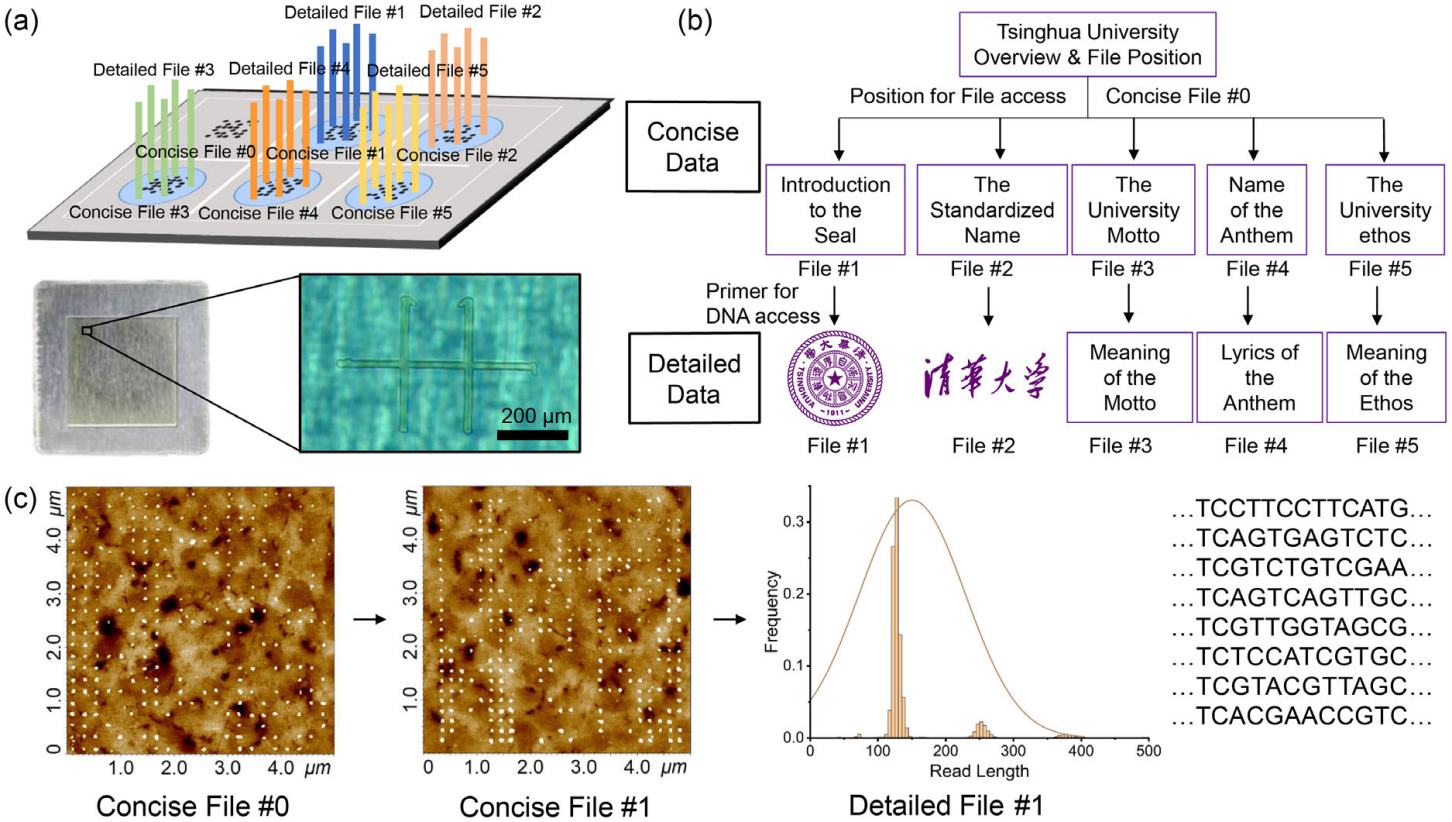
Demonstration of the dual‐mode data storage system. a,b) Illustration of storing concise and detailed data in 6 units on ITO. c) The random access process of the seal file, including the AFM images of dot arrays for concise file #0 and file #1 as well as the distribution of sequencing read length and part of the sequencing results of detailed file #1. d) Error rates of the random access sequencing results.

To verify the practical application of this dual‐mode data storage system, a library composed of five detailed files and six concise files was first constructed on an ITO substrate (**Figure** **5**a). As illustrated in Figure 5b, the concise and detailed data of seal, name, motto, anthem, and ethos of Tsinghua University were stored in five consecutive concise and detailed files, and the overview and positional information were stored in the catalog file (concise file #0). The random access of data was demonstrated by acquiring the detailed seal information in the dual‐mode data storage system (Figure 5c). First, the unit 0 corresponding to the catalog file was imaged by AFM, from which the positional information of the seal file was obtained. Next, the corresponding unit was located and imaged again by AFM, and the concise information (i.e., introduction of the seal) as well as the primer sequence were acquired. With the obtained primer information, in situ PCR was performed on unit 1. Finally, the generated DNA molecules were amplified and sequenced. The distribution of sequencing read length and part of the sequencing results of detailed file #1 (i.e., the pattern of the seal) were also shown in Figure 5c. It was observed that the majority of read lengths were located in the range of 100–150 bp, which excellently matched the designed length of DNA. Following the same procedure, other files were also successfully accessed (Figure S9–S11). or each file, 100 × sequencing results were filtered from it, and the sequencing results were filtered and aligned by two‐end primers. Then, clustering was performed by starcode, and the top n chains were selected as the clustering result (*n* = number of chains corresponding to the file), and the editing distances between the original sequences and the clustering result were compared to get the error rate (Figure 5d).

Recently, researchers have proposed a variety of storage methods that do not rely on DNA synthesis. A method based on DNA punch cards for storing data via enzymatic nicking was proposed to achieve lower cost, write latency, and error‐rates.^[^ ^35^ ^]^ The storage and reading methods based on DNA origami and super‐resolution microscopy provides a new approach to information storage that does not require DNA sequencing for data retrieval.^[^ ^36^ ^]^ Furthermore, clustered regularly interspaced short palindromic repeat (CRISPR) technology was used to modify DNA within *E. coli*, offers a novel DNA data writing approach independent of synthesis and completes data writing directly within living cells.^[^ ^37^ ^]^ CRISPR base editors are also used to edit preset DNA molecules for data writing, achieving lower costs and more efficient data writing.^[^ ^38^ ^]^ These pioneering studies have introduced innovative approaches to DNA storage, achieving better writing or reading efficiency. Compared to these works, our study continues to employ synthesis and sequencing for DNA data read and write processes. By incorporating detailed data in DNA molecules with associated concise data in nanodot array, this integrated dual‐mode storage system offers an effective method for managing multiple DNA files and enables improved multiple random reads. The system can be further developed based on the aforementioned pioneering work by combining the dual‐mode management strategy with efficient read and write methods.

## 3 Conclusion

This study proposes a dual‐mode DNA storage management system and explores the corresponding methods to make it work. Concise data writing/reading is made possible through the SPL/AFM approach, and detailed data writing/reading is achieved through DNA immobilization and in situ PCR. The concept of dual‐mode storage is further validated by a simple demo. The highly dense protruded nanodot array not only contains sufficient concise data, but also enables facile and frequent readout by efficient morphological scanning techniques. When acquiring more detailed information is needed, the corresponding primer sequence can be facilely extracted from the nanodot array, allowing for the subsequent readout of detailed data by in situ PCR and sequencing. Moreover, since DNA is immobilized on physically isolated microregions, reliable random multiplexed access can be easily achieved. Therefore, this novel storage system could accomplish efficient management of concise and detailed data, which opens a new revenue for the development of DNA‐based data storage. Future research could focus on new methodologies to fabricate nanodot arrays and immobilize DNA with the aim of enhancing data storage density, read/write speed, and lifespan.^[^ ^28,^, ^30c^ ^]^ It is believed that the advancement of writing and reading technologies could further propel this novel data storage system toward maturation and industrialization.

## 4 Experimental Section

4.1

4.1.1

##### The Preparation of Substrate and the Immobilization of DNA

To prepare the substrate, pristine ITO glass was first cleaned via the radio corporation of america (RCA) method (ammonium hydroxide: hydrogen peroxide: water = 1:4:20, v/v/v). The surface was then modified with a thin thiolated layer by vaporizing MTPS (Sigma) at room temperature in a vacuumed desiccator overnight. After removing the MTPS oligomers by ultrasonicating in an ethanol bath, the resulting SH@ITO disk was cleaned with excess ultrapure water and dried under nitrogen stream, followed by baking on a 100 °C concise plate. After the fabrication of nanodots array, the SH group was reacted with the disulfide group of ssDNA by the thiol/disulfide exchange reaction. Specifically, the ssDNA was dissolved 1 ×  TE buffer and diluted in the carbonate buffer (0.5 M, pH 9.0, Sangon Biotech). The DNA solution was then applied to the surface of SH@ITO by pipette or micro‐syringe and allowed to react for 5–30 min. The unreacted DNA was washed off by at least three cycles of alternate 10 × TNTw buffer (Sangon Biotech) and ultrapure water. The resulting DNA disk was then dried under nitrogen stream.

##### Nanodot Array Fabrication and Detection

The study employed a commercially available NT‐MDT AFM to facilitate the fabrication and identification of nanodot arrays. The experiments were carried out utilizing a W_2_C probe (HA_HR/W_2_C, ScanSens) with the frequency of 200 kHz and the elastic modulus of 12 N m^−1^. Both the fabrication and imaging of the nanodots were conducted using the tapping mode. The SH@ITO substrate was securely attached to a conductive slide using conductive tape and grounded. During the preparation of the nanodots, a bias voltage ranging from −10 to −40 V was applied to the probe for a duration of 10–500 ms. The data storage experiments were carried out in an environment with a temperature of ≈20 °C and a relative humidity of 45%. The samples were scanned by the AFM using tapping mode. The acquired images were subsequently flattened and the color contrast was adjusted in order to obtain the scanned results and binary images suitable for data retrieval.

##### Data Coding and Decoding

The concise data was encoded by Unicode, and data files were converted into square matrix consisting of 0 and 1. The encoding of detailed data was performed according to modulation coding rules in order to fulfill the criteria of GC content and homopolymer. By employing various modulation primers and conducting modulation operations, the original base sequence could be mapped onto alternative sequences. Subsequently, when coupled with filtering conditions, it became possible to identify encoding outcomes that satisfied the given constraints from a range of modulation outcomes. In the present study, the minimum length of a single sequence was 124 nucleotides (nt), and each base sequence contained at least 60 nt of valid information. Additionally, two modulation primers consisting of four nucleotides each, a four‐nucleotide index sequence, 8 nt logical redundancy added by Reed–Solomon (RS) code and a minimum of a 4 nt Seed sequence were included. Finally, PCR primers (20 nt × 2) and an 8 nt error correction sequence generated by RS encoding were appended to each sequence. In the conducted experiment, the process of encoding was carried out on a total of 404 bytes of textual information and 1595 bytes of image data. This resulted in the generation of 135 base sequences, which collectively had a length of 17 024 nt.

##### Synthesis and Sequencing of DNA

All DNA molecules were ordered from Nanjing GenScript Biotechnology Co. Ltd, and the DNA molecules that need to be immobilized on the substrate surface were modified with a disulfide bond at the 5′ end. The DNA molecules were sequenced using the nanopore technologies (Oxford MinION). Prior to sequencing, the concentration and purity of DNA samples were assessed using Nanodrop (Thermo Scientific). DNA samples were subjected to PCR to fulfill the requirement of 400 ng – 1 μg quality. Then, the construction of a DNA library was successfully accomplished by means of sample purification and fragment enrichment utilizing a sequencing kit. Finally, the DNA library was loaded onto the sequencer chip, the sequencing program was adjusted, and the sequencing time was controlled based on the amount of data.

##### In situ PCR

DNA replication was accomplished on the substrate by employing in situ PCR at room temperature, followed by extraction of solution using a micro‐pipette. The in situ PCR was conducted using the basic RT‐ERA Nucleic Acid Amplification Kit (GenDx Biotechnology, Suzhou, China). 25 μL of premix, 22 μL of ultrapure water, and 2 μL of forward primer were mixed to prepare the reaction solution. This solution was subsequently transferred onto the DNA‐immobilized substrate using a micro‐pipette. The reaction was permitted to proceed for a duration of 2 min prior to being aspirated back into the pipette. Since only the forward primer was added to the reaction mixture, only the DNA bound to the substrate underwent replication, resulting in the production of ssDNA. The ssDNA present in the reaction mixture was not replicated. Noted that the multiple access experiments were carried out with a normal pipette (2.5 μL) to ensure the accuracy of quantification.

##### DNA Quantification

We employed the iTaq Universal SYBR Green Supermix (Bio‐Rad) for our qPCR analysis. The qPCR was conducted using the CFX96 Touch Real‐Time PCR Detection System (Bio‐Rad). A 50 μL reaction solution was prepared by combining 25 μL of premixed solution, 18.5 μL of ultrapure water, 2.5 μL of the sample, and 2 μL each of 10 μM forward and backward primers. The initial denaturation step was performed at a temperature of 95 °C for a duration of 3 min. This was followed by denaturation at 98 °C for 20 s annealing at 50 °C for 15 s, and extension at 72 °C for 15 s. The entire process was repeated for a total of 40 cycles. Finally, the reaction mixture was cooled to 4 °C. Baseline correction was conducted utilizing the CFX Maestro software, and subsequently, the threshold cycle (CT) was determined. Absolute quantification of the sample was accomplished by conducting qPCR in conjunction with DNA samples of established concentrations. The sample concentration was then determined by analyzing the CT value.

##### Statistical Analysis

Data are not pre‐processed unless explicitly declared. All data is showed by mean ± SD unless explicitly declared. Sample size (*n*) is 3 for statistical analysis at Figure 2d,e, 3e, 5 for statistical analysis at Figure 2g. The normality of the data underwent Shapiro–Wilk testing, and *p* > 0.05 indicated that the original data conformed to a normal distribution. For three or more groups, statistical analysis was conducted using one‐way analysis of variance (ANOVA). Significance was defined as *p* ≤ 0.05. Data analysis and visualization were mostly performed in OriginPro 2021. Sequencing analysis were mostly performed in Ubuntu 20.04. Some tools were used for these works, which included seqkit, starcode, bowtie2, SAMtools. Sequencing data analysis and visualization were mostly performed in Spyder5.4.3 using python3.11 language. Some standard python extension packages were used for these works, which included numpy, pandas, scipy, levenshtein, sklearn, matplotlib, seaborn.

## Conflict of Interest

The authors declare no conflict of interest.

## Author Contributions

**Ben Pei**: Conceptualization (lead); Data curation (lead); Formal analysis (lead); Investigation (lead); Methodology (lead); Validation (lead); Visualization (lead); Writing—original draft (lead). **Yongsen Zhou**: Data curation (equal); Formal analysis (equal); Investigation (equal); Methodology (equal); Validation (equal); Writing—review and editing (lead). **Yu Yang**: Formal analysis (equal); Investigation (equal); Validation (equal). **Jiaxiang Ma**: Data curation (equal); Formal analysis (equal). **Rangli Cao**: Methodology (supporting); Software (equal). **Wen Huang**: Investigation (supporting); Validation (supporting). **Liliang Ouyang**: Supervision (equal). **Shengli Mi**: Supervision (equal). **Zhuo Xiong**: Funding acquisition (lead); Supervision (lead); Writing—review and editing (equal).

## Supporting information

Supplementary Material

## Data Availability

The data that support the findings of this study are available from the supporting information. The sequencing data are openly available at https://cloud.tsinghua.edu.cn/d/58cdde82169c4e7f811c/.
