# Supplementary material for: A Novel DNA‐Based Dual‐Mode Data Storage System with Interrelated Concise and Detailed Data
Source: Small Sci. 2024 Aug 19;4(11):2400094. doi: 10.1002/smsc.202400094 (PMC11935158; doi:10.1002/smsc.202400094)
Supplement: Supplementary file 1 — Supplementary Material [file SMSC-4-2400094-s001.pdf]

Supporting Information

A NOVEL DNA-BASED DUAL-MODE DATA STORAGE SYSTEM WITH INTERRELATED CONCISE AND DETAILED DATA

Ben Pei[1,2,3], Yongsen Zhou[1,2,3], Yu Yang[1,2,3], Jiaxiang Ma[4], Rangli Cao[4], Wen Huang[1,2,3], Liliang Ouyang[1,2,3], Shengli Mi[4], Zhuo Xiong[1,2,3, *]

[1] Biomanufacturing Center, Department of Mechanical Engineering, Tsinghua University, Beijing, 100084, China

[2] Biomanufacturing and Rapid Forming Technology Key Laboratory of Beijing, Beijing, 100084, China

[3] Biomanufacturing and Engineering Living Systems, Innovation International Talents Base (111 Base), Beijing, 100084, China

[4] Bio-manufacturing Engineering Laboratory, Tsinghua Shenzhen International Graduate School, Tsinghua University, Guangdong, Shenzhen, China

Note1: Calculation of data storage density

For the calculation of concise data storage density, the flat and protruding states of each nanodot respectively represent the data states 0 and 1. Hence, each nanodot position can store 1 bit of information. Consequently, the concise data storage density $D_h = d^{-2}$, where d represents the spacing between nanodots in the array. For the calculation of detailed data storage density, since DNA molecules are immobilized on a two-dimensional plane, the data storage density is determined by the average spacing between DNA molecules and the information capacity stored within the DNA molecules themselves. Assuming that the length of DNA molecules is 120 nucleotides, the primer and error correction code are about 56 nt according to the coding method in this paper, one DNA molecule can store 188 bits of information. Thus, the detailed data storage density $D_c = 188 \times d^{-2}$, where d represents the average distance between DNA molecules. The evaluation pertains to the theoretical storage density performance of the dual-mode storage system. Hence, the calculation of data storage density only considers physical factors and does not consider encoding efficiency and the impact of redundancy on storage density.

Note2: Accelerated aging experiment.

In our research, we conducted accelerated aging experiments on the storage system to measure its stability. The storage system was placed in glass bottles filled with nitrogen and subjected to accelerated aging at temperatures of 60°C, 65°C, and 70°C. After various time intervals, DNA molecules were extracted using a 2-minute solid-phase room-temperature PCR. The relative quantity of these DNA molecules over storage time is shown in Figure S4. The data was fitted using formula $c = e^{-kx}$ to obtain coefficients k at various temperatures (Table S1). Fitting was also done using the Arrhenius equation $k = Ae^{\frac{-E_A}{RT}}$ to obtain the activation energy E_A (Figure 4g). The fitted value of E_A is 83.99 kJ mol^{-1}, which is slightly lower than the typical range of DNA activation energy, which is 120-155 kJ mol^{-1}. This is reasonable because in the storage system, besides DNA degradation, the failure of the connection between DNA and the substrate can also result in storage loss.

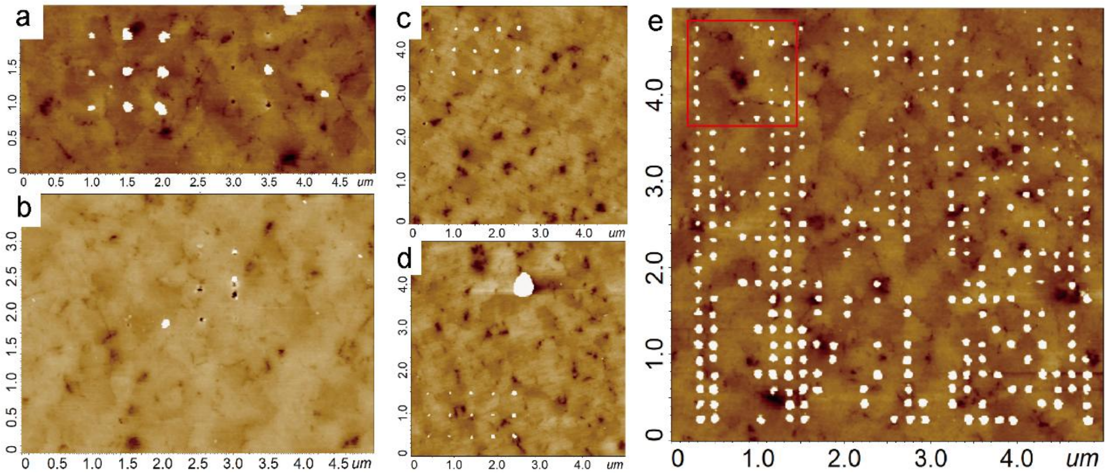

Figure S1. The lithography results under different condition. a. Lithography results under 45% RH (original data of Figure 2b1). b. Lithography results under 15% RH (original data of Figure 2b2). c. Lithography results at different voltages (original data of Figure 2d). d. Lithography results at different pulse time (original data of Figure 2e). e. Morphological characterization of the nanodot array under optimized condition (original data of Figure 2f, Figure 2f displays the area within the red rectangle).

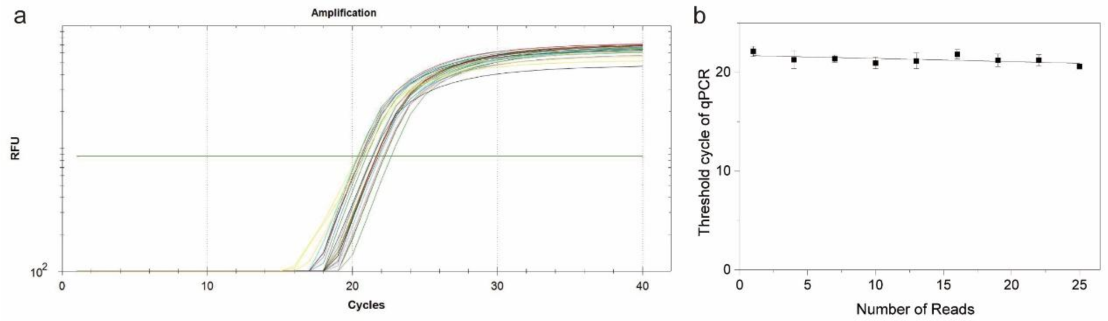

Figure S2. QPCR results of DNA extraction replicates. a. Original results of the qPCR data from the 1st, 4th, 7th, 10th, 13th, 16th, 19th, 22nd, and 25th extractions. b. The threshold cycle of qPCR for DNA molecules obtained from 25 repeated extractions. The CT values did not show an upward trend with the increase of reading times, which indicated that the quantity of DNA molecules did not decrease significantly after multiple repetitions. The weak downward trend of CT values may be due to an incomplete cleaning.

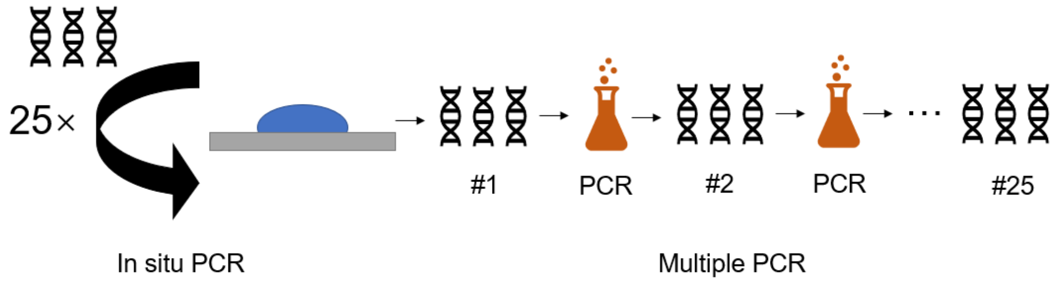

Figure S3. DNA repeat access process. The experimental group repeated the extraction process by performing in situ PCR on the substrate. The control group used the DNA initially extracted from the substrate as the initial sample and conducted multiple PCR amplifications.

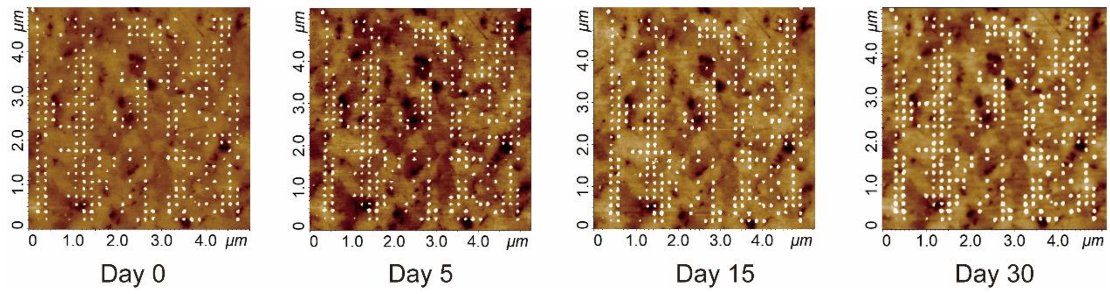

Figure S4. AFM images of nanodot arrays accessed at various days at 70 ℃. The arrays remained unchanged after 30 days of at the temperatures of 70 ℃.

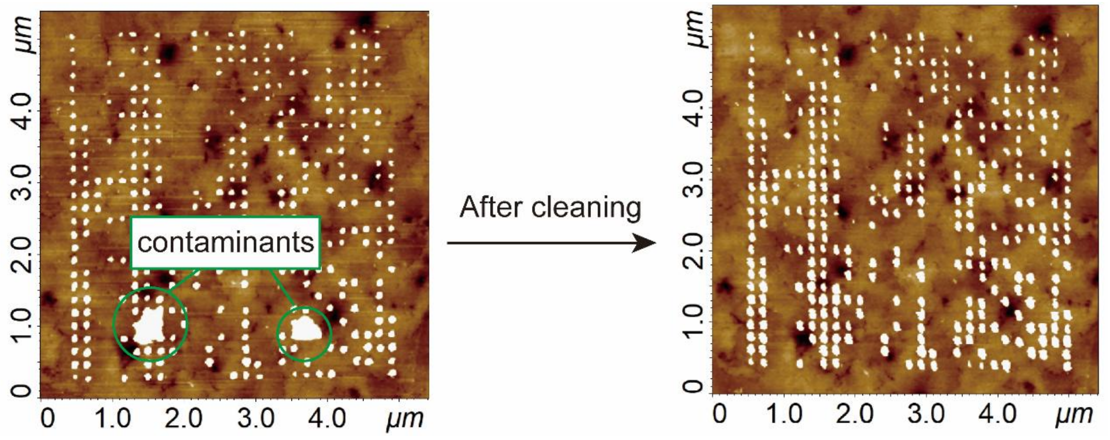

Figure S5. AFM images of nanodot arrays contaminated and after cleaning.

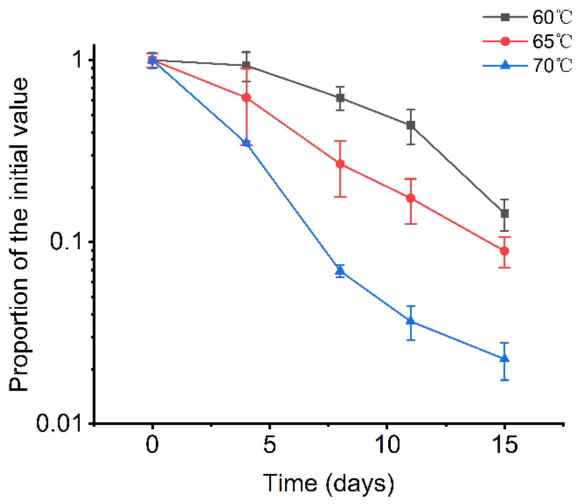

Figure S6. The experiment on accelerated aging examined the changes in the amount of DNA over time at various temperatures.

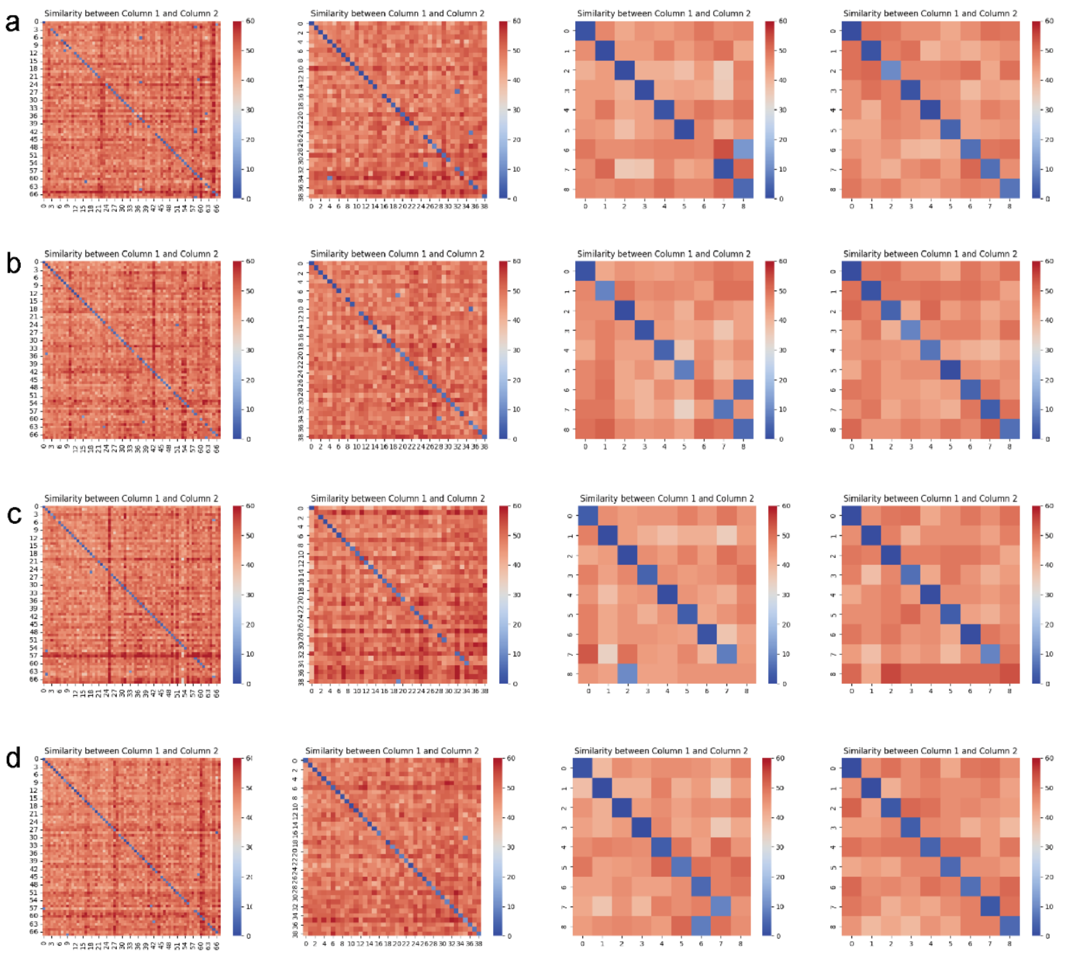

Figure S7. Clustering results of sequencing for DNA molecules in aging experiments. The blue squares on the diagonal indicate that the sequencing results were a match for the corresponding sequences. File #5 is culled because of a significant error in the results of original DNA. a. Results of original DNA. b. Results of DNA accessed after

one decay half-life at 70 ℃. c. Results of DNA accessed after one decay half-life at 65 ℃. d. Results of DNA accessed after one decay half-life at 60 ℃.

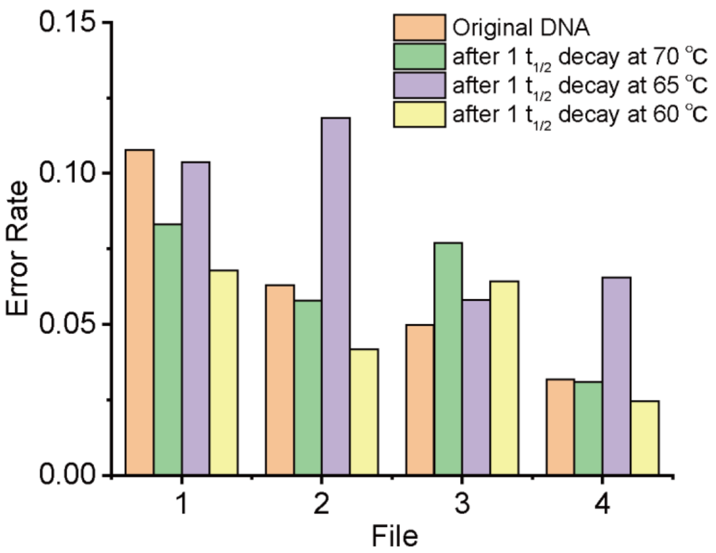

Figure S8. Error rates of the sequencing results for DNA molecules in aging experiments.

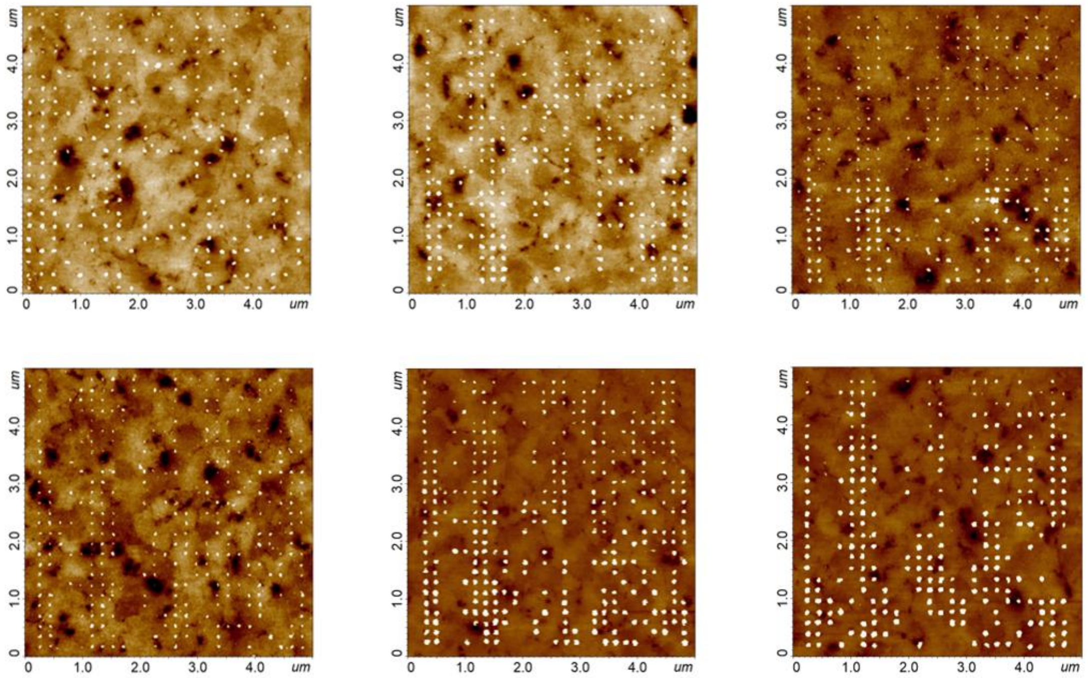

Figure S9. Results of random read access to concise data files.

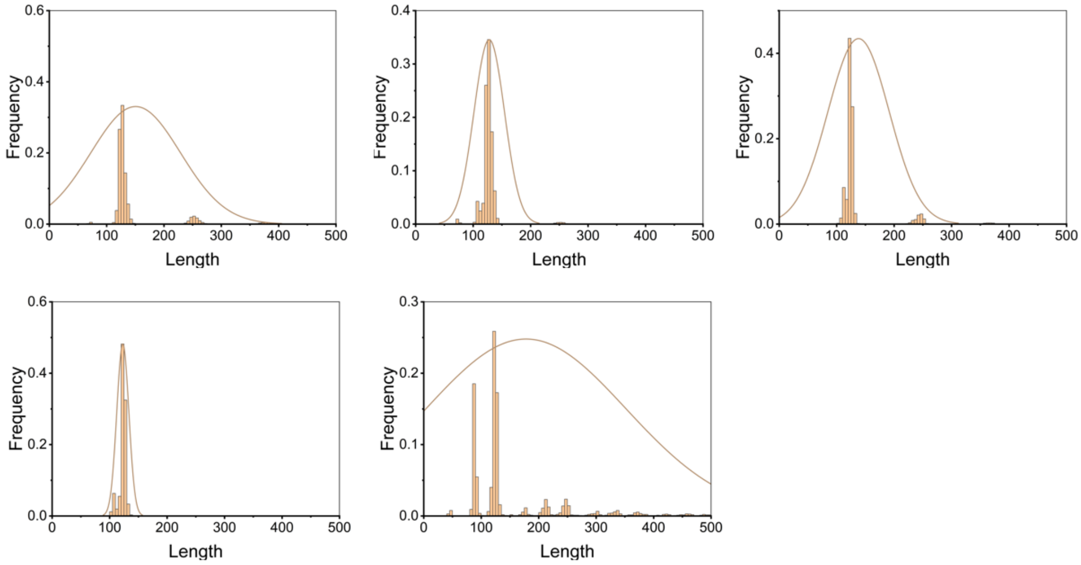

Figure S10. Random access results of detailed data files. The graphs show the distribution of read lengths for five sequencing results.

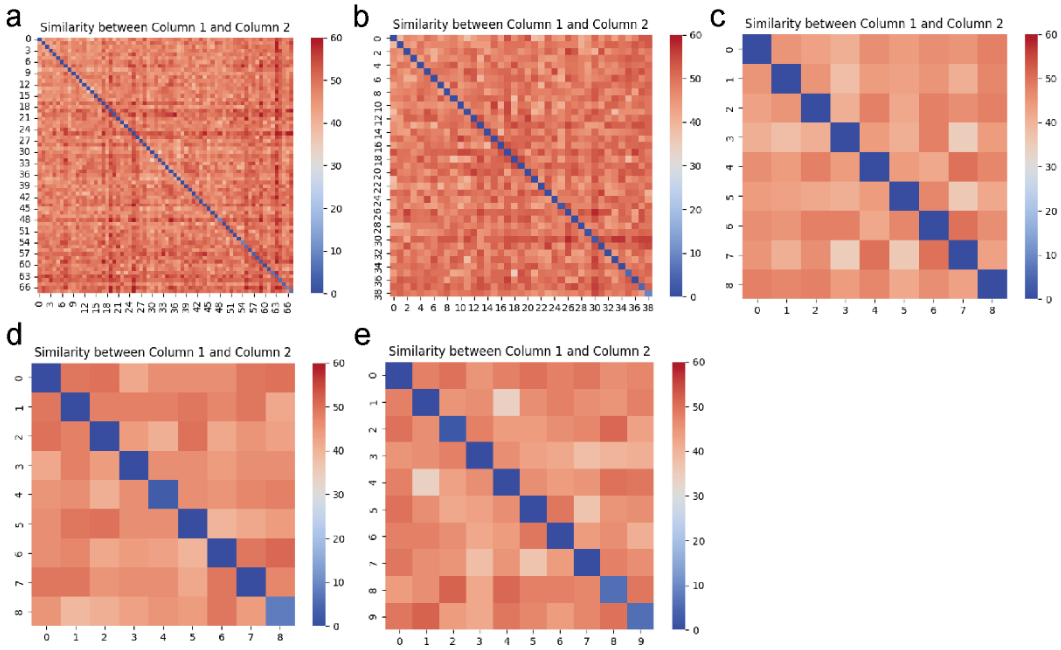

Figure S11. Clustering results of random accessed detailed data files. The graphs show the clustering analysis for five sequencing results. The blue squares on the diagonal indicate that the sequencing results were a match for the corresponding sequences.

Voltage		Point 1	Point 2	Point 3	Average	Stdev
-10V	Hight(nm)	3.523281	3.247162	5.982896	4.251113	1.506109
	FWHM(nm)	20.17364	35.09773	25.31635	26.86258	7.581243
-12V	Hight(nm)	14.23847	12.79527	8.661954	11.89856	2.894381
	FWHM(nm)	53.38006	69.48316	37.74732	53.53685	15.8685
-14V	Hight(nm)	19.64395	11.50718	10.47559	13.87558	5.022121
	FWHM(nm)	71.93574	56.09057	69.64498	65.89043	8.563866
-16V	Hight(nm)	23.96964	20.61038	14.62926	19.73643	4.731123
	FWHM(nm)	82.27926	60.13183	74.07973	72.16361	11.19736
-18V	Hight(nm)	22.65503	20.94731	19.69745	21.09993	1.484683
	FWHM(nm)	79.85122	60.81449	57.38643	66.01738	12.10245

Table S1. Original data of lithography results at different voltages.

Pulse time		Point 1	Point 2	Point 3	Average	Stdev
2ms	Hight(nm)	6.10335	4.935572	6.810123	5.949682	0.946676
	FWHM(nm)	41.25332	40.08403	29.42702	36.92145	6.516654
5ms	Hight(nm)	5.200338	7.040004	4.767516	5.669286	1.206642
	FWHM(nm)	46.99628	49.02707	33.82077	43.28137	8.255804
25ms	Hight(nm)	6.604369	7.685482	5.607147	6.632333	1.039449
	FWHM(nm)	44.0648	55.76484	41.06601	46.96522	7.766801
100ms	Hight(nm)	12.80505	10.42131	19.68426	14.30354	4.809847
	FWHM(nm)	61.26275	46.86622	75.50118	61.21005	14.31755
300ms	Hight(nm)	24.46048	20.96592	14.12931	19.8519	5.254907
	FWHM(nm)	91.83692	62.20047	89.23006	81.08915	16.40992

Table S2. Original data of lithography results at different pulse time.

	1	2	3	4	5	Average	Stdev
ITO	38.3	39.8	38.6	41.1	36.7	38.9	1.48
SH@ITO	67.9	68.7	69.2	68	67.8	68.3	0.54
DNA@ITO	59	55.9	57.1	60.2	55.9	57.6	1.72

Table S3. Original data for wetting characterization of the substrate.

Multiple reads	CT value 1	CT value 2	CT value 3	Average	Stdev
1st	22.61	22.18	21.59	22.12667	0.512087
4th	21.34	22.18	20.34	21.28667	0.921159
7th	21.62	21.57	20.96	21.38333	0.367469
10th	20.71	20.52	21.61	20.94667	0.582266
13th	21.86	20.3	21.28	21.14667	0.788501
16th	21.27	22.1	22.12	21.83	0.485077
19th	21.56	21.61	20.49	21.22	0.632693
22th	21.73	20.6	21.34	21.22333	0.573963
25th	20.82	20.29	20.64	20.58333	0.269506

Table S4. Original data for QPCR results of DNA extraction replicates.

	k[s^{-1}]	R^2
60℃	1.15E6	0.89
65℃	1.85E6	0.99
70℃	2.19E6	0.95

Table S5. Results of fitting at different temperatures. Fitted using linear fitting with intercept set to 0. (Ln(c) vs. time)
